# Clinical and imaging outcomes after intrathecal injection of umbilical cord tissue mesenchymal stem cells in cerebral palsy: a randomized double-blind sham-controlled clinical trial

**DOI:** 10.1186/s13287-021-02513-4

**Published:** 2021-08-06

**Authors:** Man Amanat, Anahita Majmaa, Morteza Zarrabi, Masoumeh Nouri, Masood Ghahvechi Akbari, Ali Reza Moaiedi, Omid Ghaemi, Fatemeh Zamani, Sharif Najafi, Reza Shervin Badv, Massoud Vosough, Amir Ali Hamidieh, Mona Salehi, Hadi Montazerlotfelahi, Ali Reza Tavasoli, Morteza Heidari, Hossein Mohebi, Ali Fatemi, Amir Garakani, Mahmoud Reza Ashrafi

**Affiliations:** 1 Department of Science and Research Branch, AJA University of Medical Sciences, Tehran, Iran; 2 Pediatrics Center of Excellence, Department of Pediatric Neurology, Children’s Medical Center, Growth and Development Research Center, Tehran University of Medical Sciences, Tehran, Iran; 3 Department of Regenerative Medicine, Cell Science Research Center, Royan Institute for Stem Cell Biology and Technology, ACECR, Tehran, Iran; 4 Department of Pediatric Neurology, Clinical Research Development Center of Children Hospital, Hormozgan University of Medical Sciences, Bandar Abass, Iran; 5 Pediatrics Center of Excellence, Department of Radiology, Children’s Medical Center, Tehran University of Medical Sciences, Tehran, Iran; 6 Clinical Biomechanics and Ergonomics Research Center, Department of Physical Medicine and Rehabilitation, Faculty of Medicine, AJA University of Medical Sciences, Tehran, Iran; 7 Pediatrics Center of Excellence Pediatric Hematology, Oncology and Stem Cell Transplantation Department, Children’s Medical Center, Tehran University of Medical Sciences, Tehran, Iran; 8 Psychiatry and Psychology Research Center, Tehran University of Medical Sciences, Tehran, Iran; 9 Department of Pediatrics, Faculty of Medicine, Tehran Medical Sciences, Islamic Azad University, Tehran, Iran; 10 Department of Pediatric Neurology, AJA University of Medical Sciences, Tehran, Iran; 11 Moser Center for Leukodystrophies, Kennedy Krieger Institute, Baltimore, MD 21205 USA; 12 Department of Neurology and Pediatrics, Johns Hopkins University School of Medicine, Baltimore, MD 21287 USA; 13 Department of Psychiatry, Yale School of Medicine, New Haven, CT USA; 14 Department of Psychiatry, Icahn School of Medicine at Mount Sinai, New York, NY USA

**Keywords:** Cerebral palsy, Stem cell, Diffusion tensor imaging, Gross motor function, Children

## Abstract

**Background:**

This study assessed the safety and efficacy of intrathecal injection of umbilical cord tissue mesenchymal stem cells (UCT-MSC) in individuals with cerebral palsy (CP). The diffusion tensor imaging (DTI) was performed to evaluate the alterations in white-matter integrity.

**Methods:**

Participants (4–14 years old) with spastic CP were assigned in 1:1 ratio to receive either UCT-MSC or sham procedure. Single-dose (2 × 10^7^) cells were administered in the experimental group. Small needle pricks to the lower back were performed in the sham-control arm. All individuals were sedated to prevent awareness. The primary endpoints were the mean changes in gross motor function measure (GMFM)-66 from baseline to 12 months after procedures. The mean changes in the modified Ashworth scale (MAS), pediatric evaluation of disability inventory (PEDI), and CP quality of life (CP-QoL) were also assessed. Secondary endpoints were the mean changes in fractional anisotropy (FA) and mean diffusivity (MD) of corticospinal tract (CST) and posterior thalamic radiation (PTR).

**Results:**

There were 36 participants in each group. The mean GMFM-66 scores after 12 months of intervention were significantly higher in the UCT-MSC group compared to baseline (10.65; 95%CI 5.39, 15.91) and control (*β* 8.07; 95%CI 1.62, 14.52; Cohen’s *d* 0.92). The increase was also seen in total PEDI scores (vs baseline 8.53; 95%CI 4.98, 12.08; vs control: *β* 6.87; 95%CI 1.52, 12.21; Cohen’s *d* 0.70). The mean change in MAS scores after 12 months of cell injection reduced compared to baseline (−1.0; 95%CI −1.31, −0.69) and control (*β* −0.72; 95%CI −1.18, −0.26; Cohen’s *d* 0.76). Regarding CP-QoL, mean changes in domains including friends and family, participation in activities, and communication were higher than the control group with a large effect size. The DTI analysis in the experimental group showed that mean FA increased (CST 0.032; 95%CI 0.02, 0.03. PTR 0.024; 95%CI 0.020, 0.028) and MD decreased (CST −0.035 × 10^-3^; 95%CI −0.04 × 10^-3^, −0.02 × 10^-3^. PTR −0.045 × 10^-3^; 95%CI −0.05 × 10^-3^, −0.03 × 10^-3^); compared to baseline. The mean changes were significantly higher than the control group.

**Conclusions:**

The UCT-MSC transplantation was safe and may improve the clinical and imaging outcomes.

**Trial registration:**

The study was registered with *ClinicalTrials.gov* (NCT03795974).

**Supplementary Information:**

The online version contains supplementary material available at 10.1186/s13287-021-02513-4.

## Introduction

Stem cells are defined as pluripotent cells with the ability of self-renewal and the capacity of differentiation into the other cell types. There has been greater interest in the use of stem cell therapy in recent years; especially for the treatment of neurological disorders [1]. The central nervous system (CNS) is unable to regenerate new cells and damages to CNS can be permanent. Several studies assessed the safety and efficacy of different stem cells in the treatment of individuals diagnosed with stroke [2], multiple sclerosis [3], Parkinson’s disease [4], Huntington’s disease [5], and spinal cord injury [6]. To date, many aspects of cell-based therapy remained unknown. The optimal dose, the most appropriate type of cell, and the best route of cell administration should be identified to provide safe and effective protocols without raising ethical concerns. Different underlying mechanisms of action have been described to justify the potential efficacy of stem cell therapy. Regarding mesenchymal stem cells (MSCs), it is believed that paracrine signaling and immunomodulation have the most critical effects. These cells can release neurotrophic factors, anti-oxidant molecules, angiogenic, anti-inflammatory, anti-fibrotic, and anti-apoptotic agents that enhance tissue repair after injury [7, 8]. The capacity of MSCs to regenerate and differentiate to new cells is another proposed mechanism [9] but studies reported its limited efficacy and showed that migration of cells to the site of injury is not necessary [7]. It is now clear that the mechanism of action for many stem cells is not a consequence of differentiation [10].

The umbilical cord derives from the yolk sac and contains two arteries, one vein, and a gelatinous substance composed of sulfated proteoglycans with collagenous fibers; known as Wharton’s jelly [11]. The umbilical cord was found to have great proportions of stem cells. The first successful stem cell transplant was from umbilical cord blood cells on a 6-year-old-boy with Fanconi anemia in 1988 [12]. Low immunogenicity, low risk of graft versus host disease, and ease of cell collection are major advantages of using cells derived from the umbilical cords [11]. Umbilical cord tissue mesenchymal stem cell (UCT-MSC) has been used in recent studies to determine their safety and clinical efficacy [13–15].

Cerebral palsy (CP) is the leading cause of physical disability in children and is known as a group of non-progressive permanent CNS disorders that affected movements, muscle tone, and coordination [16]. The global prevalence of CP was estimated to be up to 3 per 1000 individuals [17]. Preclinical studies reported that some types of stem cells (e.g., MSC) had neuroprotective effects on animal models of neonatal hypoxia-ischemia [18–20]. Few randomized trials demonstrated the promising clinical effects of stem cell therapy in children with CP (reviewed in [21–23]). We conducted this randomized double-blind sham-controlled trial to assess the safety and clinical effects of intrathecal injection of UCT-MSC in CP. To assess the impact of cell therapy on the alteration of white matter integrity, we performed quantitative diffusion tensor imaging (DTI) before and after treatment. DTI is a non-invasive imaging method that can characterize the micro-structural changes in white matter tracts based on the diffusion of water molecules. We hypothesized that the UCT-MSC could significantly improve clinical and imaging outcomes compared to the control group (superiority trial).

## Methods

### Study design

This multi-center, population-based randomized double-blind sham-controlled trial with single intrathecal stem cell injection was conducted in Children’s Medical Center and Imam Reza hospital in Tehran province and Bandar Abbas pediatric hospital in Bandar Abbas province, Iran. The study was divided into four phases: [1] initial screening phase, [2] baseline phase, [3] double-blind treatment phase, and [4] follow-up phase.

### Inclusion and exclusion criteria

Males and females aged 4 to 14 years old who were diagnosed with spastic CP according to standard criteria [16], gross motor function classification system (GMFCS) level 2–5, and white matter lesions in brain magnetic resonance imaging (MRI) (e.g., periventricular leukomalacia) were included in the study. Individuals with other types of CP (e.g., athetoid, ataxic, or mixed CP), co-morbid neurological disorders (e.g., untreated epilepsy), or congenital infections (TORCH Syndrome) were excluded. Severe anemia (hemoglobins< 8 mg/dl), coagulation disorders, history of malignancy, prior cell infusion, renal insufficiency, and liver failure were other exclusion criteria.

The ethics committee of Tehran University of Medical Sciences approved the final methods (Number: IR.TUMS.VCRREC.1996.2506). Parents of participants had access to all information of the trial and a printed protocol of the study was given to them. They were informed that participation was optional, and withdrawal was possible whenever they asked for. Written informed consent was achieved from parents before the initiation of any study procedures. We also explained the protocol to children and assent was obtained. The study was registered with *ClinicalTrials.gov* (NCT03795974) and Iranian registry of clinical trials; *irct.ir* (IRCT201706176907N13).

### Rehabilitation therapy

All individuals were under rehabilitations during the study. One approach and technique of rehabilitation was established and conducted for included participants to reduce confounders. The Bobath concept was used in this study that aimed to affect muscle tone and improve the postural alignment by specific handling techniques [24, 25]. Each session lasted for 75 min and participants attended three times per week for rehabilitation during the study.

### Randomization and blinding

All included participants were randomly assigned in 1:1 ratio using permuted block randomization via interactive web response system to receive either UCT-MSC or sham procedure, respectively. The responsible statistician was masked to the clinical data of cases. Personnel staff responsible for cell preparations was not masked, but they had no contacts with participants, parents, or investigators. They also had no information about the clinical and imaging characteristics of patients. All participants, their parents, and investigators were blinded during the study unless serious adverse events occurred that emergent evaluations and treatments by medical staff were essential. A small needle prick to the lower back was performed as a sham procedure. All individuals were sedated to prevent awareness and to reduce spasticity during the procedure.

### Procedures

#### Initial screening and baseline phases

Physical and neurological examinations were performed on children and adolescents with CP. GMFCS was used for initial functional assessment and brain MRI was also performed. Screening tests included blood count (e.g., hemoglobin, white blood cells, and platelets), serum chemistry (e.g., liver function test, creatinine, and urea), prothrombin time, partial thromboplastin time, and electro-encephalography (EEG) (phase 1). Gross motor function measure (GMFM)-66, modified Ashworth scale (MAS), pediatric evaluation of disability inventory (PEDI), and CP quality of life (CP-QoL) were used to evaluate the baseline clinical characteristics of eligible participants. Diffusion tensor imaging (DTI) was also performed to track white matter abnormalities in motor fibers (phase 2).

#### Motor function assessment

GMFCS is a 5-level clinical classification system to describe the motor function of people with CP [26, 27]. The distinctions between levels are based on functional abilities (Supplement 1). A prior study reported excellent reliability of GMFCS for children aged 2 to 12 years old with CP (kappa: 0.75) [26]. The GMFM-88 was developed to measure changes in gross motor function over time or with treatment in people with CP [28]. The GMFM-66 was found through Rasch analysis to best describe the gross motor function of children with CP of varying abilities and is a 66 item subset of the original 88 items [29]. It has a unidimensional scale providing interval scaling rather than the ordinal scaling of the GMFM-88. It was shown that inter-rater reliability of Farsi version of this scale for all dimensions was between 0.97 and 0.99 and the intra-rater reliability was 0.99 [30]. Cronbach’s alpha coefficient for all dimensions was between 0.78 and 0.94 [30].

#### Spasticity assessment

The MAS can be determined according to the examination of muscle tone [31] (Supplement 2). The scores are measured based on the level of resistance during the passive movement of the antagonist muscles. The elbow flexor, wrist flexor, knee extensor, hip adductor, and ankle plantar flexor were examined on the spastic side(s), and the mean MAS score was recorded in each individual. Participants were in sitting position to examine hip adductor and supine position to test other muscles. The interpretation of MAS should be with extreme caution. Several limitations to MAS were described previously [32]. Low inter- and intra-rater reliability of MAS in children with CP were estimated [33, 34], and the validity of the scale was poor [35]. The scale, however, quantify muscle tone in ordinal numbers and can be easily used in clinical settings.

#### Disability assessment

PEDI was developed to assess the performances of children with disabilities in 3 dimensions including self-care, mobility, and social function. The Farsi version of PEDI in children with CP was reported to have high internal consistency (Cronbach’s alpha 0.94 to 0.98). The results of test-retest reliability were excellent in self-care (0.99) and social performance [1], and good in mobility dimension (0.66) [36].

#### Quality of life assessment

CP-QoL was designed to evaluate the well-being across different domains of life in children and adolescents with CP. The CP-QoL-child form with primary caregiver proxy report was used in this trial. The domains included family and friends, participation in activities, communication, physical health, special equipment, pain and bother, access to services, and family health. Good internal consistency (Cronbach’s alpha 0.61 to 0.87) and moderate to good test-retest reliability (0.47 to 0.84) in all domains were reported in the Farsi version of questionnaire [37].

#### Brain imaging

MRI was performed on 1.5T scanner (Philips Ingenia, Eindhoven, the Netherlands). All individuals had to be remained still and cover their ears with sponge earplugs for hearing protection. Intravenous propofol (2 mg/kg dose) was used for sedation if participants did not cooperate and moved during imaging process. Intravenous thiopental (5 mg/kg dose) was used if there was sensitivity history to propofol. Possible side effects of propofol or thiopental were explained to the parents, and informed consent was obtained before using the medications. Heart and respiratory rates as well as oxygen saturation were monitored before and during the procedure. The protocol of MRI included 3D T1-weighted imaging (TR: 9.5 ms, TE: 4.6 ms, flip angle: 8 °, FOV: 210 × 210 mm^2^, voxel size: 1 × 1 × 1 mm^3^) and 2D T2-weighted sequence (TR: 4000 ms, TE: 110 ms, flip angle: 90 °, FOV: 230 × 230 mm^2^, voxel size: 0.8 × 0.8 × 3.5 mm^3^). The 2D T2-weighted sequence was used to acquire DTI data (TR: 4228 ms, TE: 94 ms, flip angle: 90 ^o^, FOV: 224 × 224 mm^2^, voxel size: 2.5 × 2.5 × 2.5 mm^3^).

#### Image post-processing

DTI processing was performed using ExploreDTI software [38]. Post-processing included a cubic interpolation and robust estimation of tensors to correct for subject motion, eddy current, and EPI distortion. Non-rigid registration on the structural images was also performed. A whole-brain white matter tract construction was carried out for each participant using a linear interpolation. Seed point resolution was set at 1 mm × 1 mm × 1 mm with a seed fractional anisotropy threshold of 0.2 and an angle threshold of 50°.

**Fig. 1:**
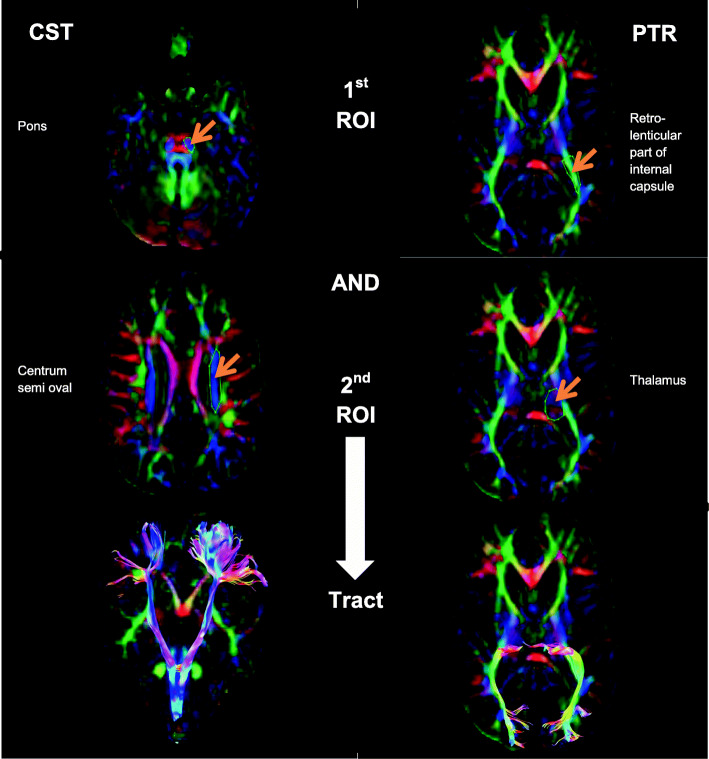
The ROI-based tractography of the corticospinal tract and posterior thalamic radiation

Region of interest (ROI)-based tractography was performed. Predefined tracts such as corticospinal tract (CST) and posterior thalamic radiation (PTR) were isolated in both hemispheres. For segmentation of CST, first ROI “AND” was drawn at the pons level and the second ROI “AND” was drawn at the centrum semi oval level. To reject the fibers that project to the cerebellum via the middle cerebellar peduncle, ROI “NOT” was drawn. For illustration of PTR, first ROI was drawn at retro-lenticular part of the internal capsule and the second ROI was at the thalamus using “AND” operation. All other tracts that not related to the PTR and were outside to ROIs were eliminated by ROI “NOT” (Fig. 1). The mean value of fractional anisotropy (FA) and mean diffusivity (MD) were, then, estimated for each separated tract in both hemispheres. The two hemispheres of each participant were compared to each other, and data of the most affected tracts were used in the analysis.

#### Cell preparation

Allogenic UCT-MSCs were derived from umbilical cord tissues of unrelated donors. The ethics committee of Tehran University of Medical Sciences approved the methods (Number: IR.TUMS.VCRREC.1996.2506). The donors were selected from full-term healthy mothers who had normal vaginal delivery without complication. The written informed consents were provided to use the umbilical cords for medical research purposes. The blood samples of donors were collected and tested for reactive transmissible infectious agents including human immunodeficiency virus, hepatitis B virus, hepatitis C virus, and cytomegalovirus. After birth, the umbilical cords were detached from the placenta and put in the plates containing phosphate-buffered saline and streptomycin. They were transferred to the laboratories of Royan at 4^o^C within 24 h. After removal of the umbilical arteries and vein, the umbilical cords were cut into 2 to 4 cm pieces to obtain Wharton’s jelly. The Wharton’s jelly was cut into 1 mm^3^ fragments. Collagenase and hyaluronidase were, then, added. The UCT-MSCs were isolated by centrifugation and cultivated in DMEM (Dulbecco’s modified Eagle’s medium) supplemented with 10% fetal bovine serum, 100 U/mL penicillin, 100 mg/mL streptomycin, and 2 mmol/L l-glutamine and, then, incubated at 37^o^C in a humidified tissue culture incubator in 5% CO_2_ and 95% air. The cells were passaged and collected at the fourth generation. Strict quality controls were performed before any clinical application. The products were analyzed for bacterial contamination using a BACTEC instrument (BD Bactec; BD Diagnostics, Franklin, NJ, http://www.bd.com). Furthermore, the amount of bacterial endotoxin was determined by limulus amebocyte lysate (LAL) kit (Lonza, Switzerland) and an ELISA reader (Amersham, USA). The cultivated cells were also tested to detect mycoplasma species by nested PCR, and karyotyping was performed to identify any chromosomal abnormalities. These procedures were conducted according to recommendations for cell and tissue therapy promotion and validation tests of the Iranian Health Ministry Pharmacopoeia Commission and the Department of Health and Human Services Food and Drug Administration.

**Fig. 2:**
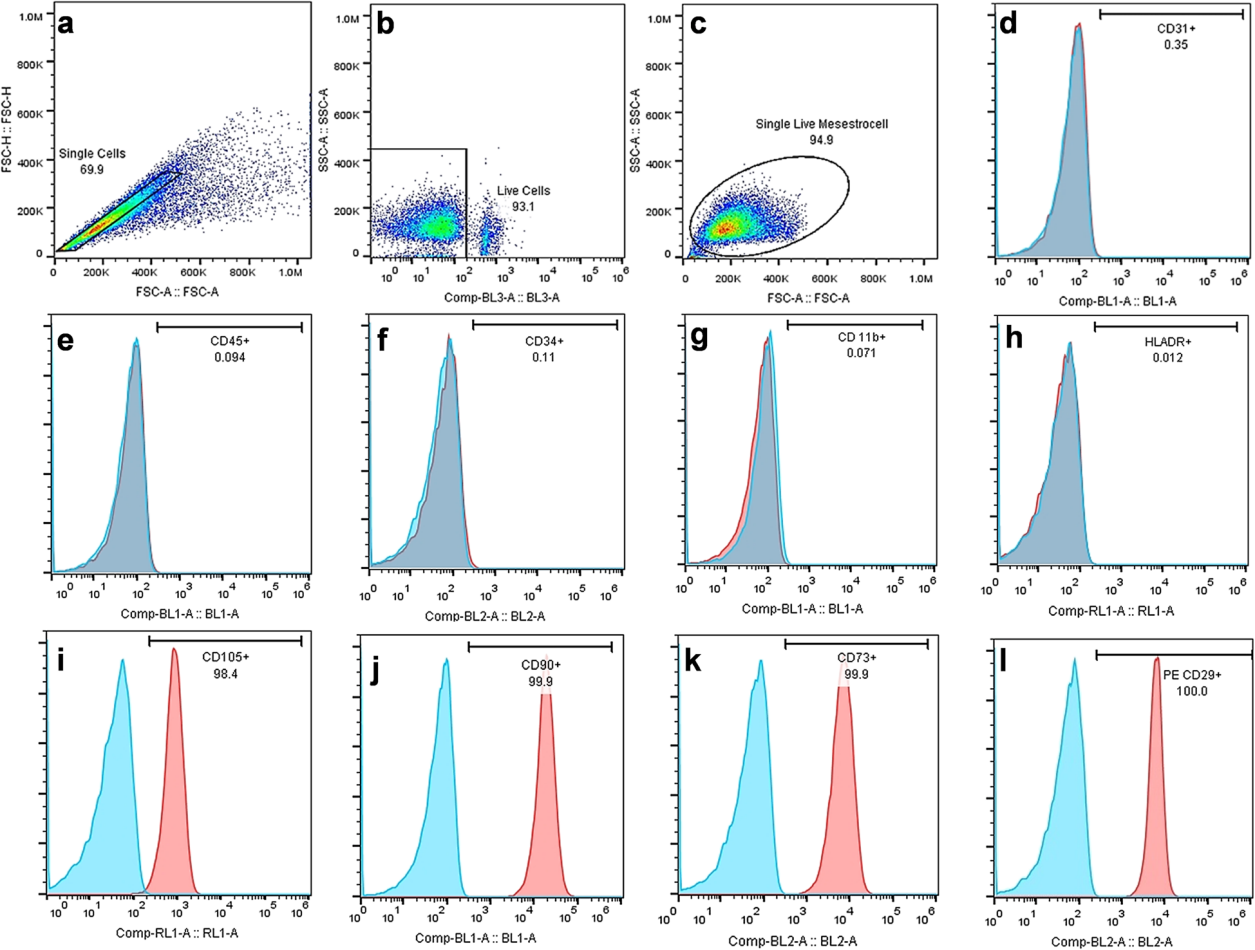
The flow cytometry analysis of UCT-MSC-specific markers. The first three photos (**a**, **b**, **c**) shows the cell gating to select single live UCT-MSCs. Othe photos (**d**-**l**) shows that the cells were negative for CD11b, CD31, CD34, CD45, and HLA-DR and were positive for CD29, CD73, CD90, and CD105

The monoclonal antibodies against the cell surface markers CD11b, CD29, CD31, CD34, CD45, CD73, CD90, and CD105, as well as isotype control antibodies (eBioscience) were used to stain the UCT-MSCs for 1 h, and the analysis was performed using FACSCalibur (BD Biosciences). The flow cytometry analysis of UCT-MSCs was shown in Fig. 2.

#### Double-blind treatment and follow-up phases

Single dose of 2 × 10^7^ cells were injected via intrathecal route in the experimental group. Cell suspensions were filtered through a cell strainer and transferred into storage vials. The cells were suspended in normal saline by unmasked personnel staff and then transferred for injection. Participants lied down on their sides (lateral decubitus position) with their knees drawn up to their chests. All participants were sedated and lumbar puncture was performed with 21 or 22 gauge spinal needles between lumbar level 2 and 5 intervertebral space after washing the back with iodine. The specific lumbar level was determined individually for each participant based on anatomical consideration. After assurance of placing the spinal needle in sub-arachnoid space, 2 mL of cerebrospinal fluid (CSF) was collected and 1 mL was sent to determine baseline CSF characteristics. The 2 mL of cells were, then, injected slowly within 2 min. The 1 mL of remained CSF was infused in the last step and after cell injection. Sham procedure was performed in the control group. The puncture site was covered with a band-aid in all individuals (phase 3). All participants were hospitalized in child neurology departments. They were placed in 10^o^ trendelenburg position to maximize the distribution of cells in CSF. No immunosuppressive medications were used. Heart rates, temperature, blood pressure, and respiratory rates were monitored for 24 h after the intrathecal injection, and individuals were discharged if there were no side-effects. The GMFM-66 and MAS were assessed 1, 3, 6, and 12 months after the intervention. The PEDI and CP-QoL were evaluated 6 and 12 months after the procedure. MRI and DTI were also performed 12 months after treatment (phase 4).

### Outcomes

Primary endpoints were the mean changes in GMFM-66 scores from baseline to 12 months after double-blind treatment phase. The mean changes in MAS, PEDI, and CP-QoL domains were also evaluated. The secondary endpoints were the mean changes in FA and MD of CST and PTR fibers from baseline to 12 months after the double-blind treatment phase. The differences between groups were also assessed.

Safety endpoints were the adverse events. All participants and their parents were asked to report any side effects during the follow-up visits. A phone number was given to the parents so adverse events could be reported as soon as possible. Parents were asked to bring their children to the emergency department if any serious complication occurred.

### Statistical analysis

Sample size was estimated based on the mean changes in GMFM-66 scores. It was calculated using repeated measures analysis of variance (ANOVA) by G*Power 3.1 software (University of Kiel, Germany). Effect size of 0.25, two-sided α (the probability of type I error) of 0.05, and β (the probability of type II error) of 0.20 were considered, and total sample size of 72 individuals was estimated after considering 10% drop-out rate to provide at least 80% power (Supplement 3).

The statistician was blinded to study groups. Numeric variables were reported as means with standard deviation (SD) or standard error of the mean (SEM). Categorical variables were presented as percentages and were compared between groups using Pearson’s chi-squared test (gender, type of CP, and GMFCS). Kolmogrov-Smirnov test was performed to report the distribution of variables. Two sided significance (*P* value) lower than 0.05 showed the non-normal distribution (GMFM-66, PEDI, and CP-QoL) and higher than 0.05 represented normal distribution of data (MAS and ROI-based data). Intention to treat approach was used and all participants who were randomized were included in the statistical analysis. Multiple imputation was conducted using Markov chain Monte Carlo to handle missing data. Generalized estimating equations (GEE) model was used to compare GMFM-66, PEDI, CP-QoL, and MAS mean scores between groups [39]. It was assumed that the interaction was between the intervention groups and time measurements. Exchangeable structure was considered for working correlation matrix and linear model was used. The model was adjusted to covariates including type of CP, GMFCS, gender, age, and weight of participants. Independent sample *t* test was conducted to compare numeric variables in baseline and DTI data between groups. Statistical analyses were performed using the IBM SPSS Software, version 25.0 (SPSS Inc., Chicago, IL) and GraphPad Prism version 7.04. Two-sided significance testing was conducted, and *P* values< 0.05 were considered statistically significant. Cohen’s *d* test with 95% confidence interval (CI) was used to measure the effect sizes that were classified as small (*d* 0 to 0.20), medium (*d* 0.20 to 0.50), and large (*d*> 0.50) using R statistical package (R Core Team, 2013).

## Results

### Participants

**Fig. 3:**
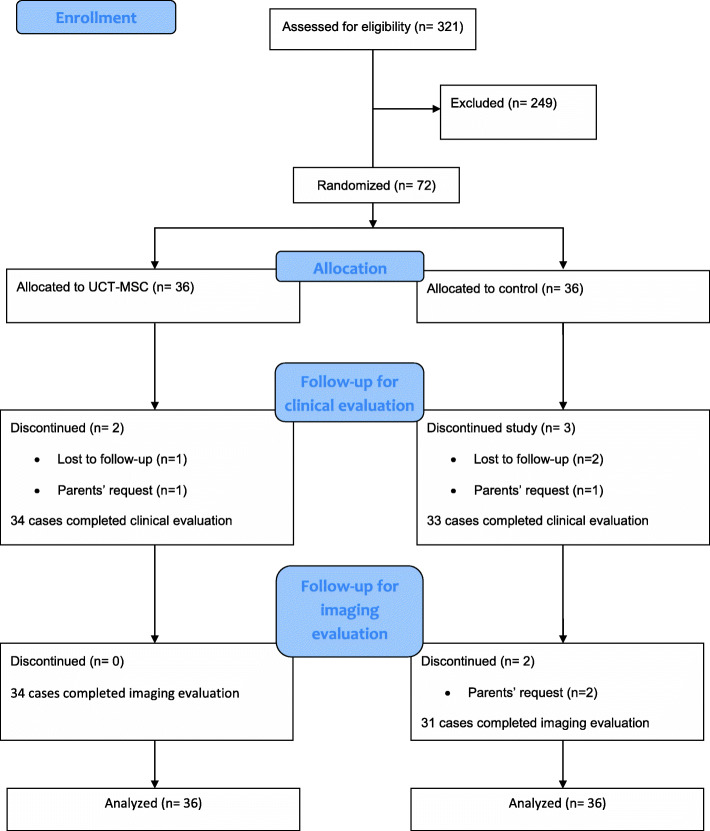
CONSORT flow diagram

We used consolidated standards of reporting trials (CONSORT) to improve our study (Supplement 4). Initial screening started on July 23, 2017. The first participant was assigned to study group on August 19, 2017, and the double-blind treatment phase lasted until November 24, 2018. The follow-up phase lasted until December 2, 2019. Primary screening to identify eligible participants was performed on 321 individuals, and 72 cases were randomly assigned to study arms (36 cases in each group). There were 5 cases (6.9%) who discontinued the study due to the lost to follow-up (*n*= 3 or 4.1%) or withdrawal of consent (*n*= 2 or 2.8%). Two participants (2.7%) were examined during the follow-up periods, but the second imaging study was not conducted due to the parents’ request (Fig. 3). Data showed that there were no differences between groups regarding the baseline demographic data (Table 1).

**Table 1:** Baseline demographic characteristics

Demographics	Control *n*=36	UCT-MSC *n*=36	*P* value
Gender *N* (%)			
Female	17 (47.2)	15 (41.7)	0.339
Male	19 (52.8)	21 (58.3)	
Age (months)			
Mean (SD)	102.5 (29.9)	101.7 (32.1)	0.186
Weight (kg)			
Mean (SD)	17.3 (7.2)	17.5 (8.5)	0.30
Type of cerebral palsy			
Spastic quadriplegia	32 (88.9)	30 (83.3)	0.491
Spastic diplegia	4 (11.1)	6 (16.7)	
GMFCS, *N*			
II/III/IV/V	4/4/11/17	5/7/10/14	0.876
GMFM-66 score			
Mean (SD)	66.3 (50.7)	70.2 (45.0)	0.769
Modified Ashworth scale			
Mean (SD)	3.16 (0.97)	2.91 (1.12)	0.215
PEDI-Self-care			
Mean (SD)	21.77 (16.15)	24.37 (17.09)	0.518
PEDI-Mobility			
Mean (SD)	16.07 (14.01)	17.53 (11.98)	0.682
PEDI-Social function			
Mean (SD)	26.63 (18.76)	32.08 (20.33)	0.243
CP-QOL			
Mean (SD)	369.8 (56.2)	364.4 (46.1)	0.644
FA corticospinal tract			
Mean (SD)	0.433 (0.63)	0.448 (0.40)	0.371
FA posterior thalamic radiate			
Mean (SD)	0.341 (0.40)	0.354 (0.32)	0.364
MD corticospinal tract^*^			
Mean (SD) ×10^3^	0.967 (0.07)	0.972 (0.06)	0.271
MD posterior thalamic^*^ radiate			
Mean (SD) ×10^3^	1.060 (0.12)	1.067 (0.07)	0.943

*SD* standard deviation, *GMFCS* growth motor classification system, *GMFM* growth motor function measurement, *PEDI* pediatric evaluation of disability inventory, *CP-QOL* cerebral palsy quality of life child, *FA* fractional anisotropy, *MD* mean diffusivity

*The means (SD) should be divided by 1000

### Primary endpoint

**Fig. 4:**
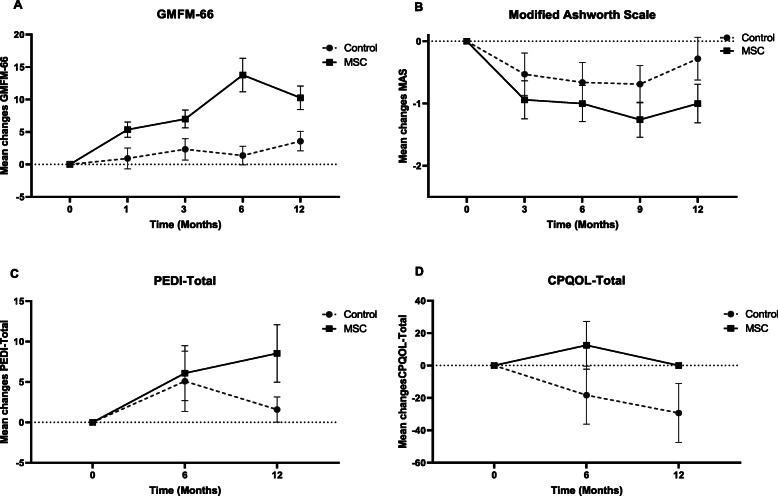
Primary endpoint analysis

We included 72 participants in the analysis. The mean change in GMFM-66 scores from baseline to 12 months after intervention was statistically significant in the UCT-MSC group (10.65, 95%CI 5.39 to 15.91) but not in control arm (1.23, 95%CI −3.33 to 5.80) compared to baseline (Fig. 4A). The mean change was statistically higher in the experimental group compared to control arm with large effect size (*β* 8.07, 95%CI 1.62 to 14.52, Cohen’s *d* 0.92) (Table 2). The assessment of other scales showed that mean MAS scores from baseline to 12 months after cell injection decreased significantly (mean change −1.0, 95%CI −1.31 to −0.69) (Fig. 4B), and the mean change in the UCT-MSC group was statistically higher than control arm with large effect size (*β* −0.72, 95%CI −1.18 to −0.26, Cohen’s *d* 0.76) (Table 2).

**Table 2:** The GMFM-66 and MAS mean difference within-groups (from baseline) and the difference between groups

Outcomes	Test of within-group (mean change from effects of baseline)		Test of between-groups effects (mean change from the control group)			
	Control	MSC	MSC vs control			
	Mean [95% CI]	Mean [95% CI]	***β***	95% CI	P.v	Cohen’s ***d*** [95% CI]
GMFM-66						
T1	−0.02 [−4.79, 4.75]	4.47 [−0.73, 9.68]	4.59	[−1.16, 10.35]	0.11	0.45 [−0.01, 0.92]
T2	2.03 [−2.97, 7.04]	**6.85 [1.49, 12.20]**	4.81	[−0.23, 9.86]	0.062	0.48 [−0.01, 0.93]
T3	−0.58 [−5.32, 4.15]	**11.27 [6.01, 16.53]**	**11.90**	**[5.42, 18.37]**	**<0.001**	1.11 [0.54, 1.67]
T4	1.23 [−3.33, 5.80]	**10.65 [5.39, 15.91]**	**8.07**	**[1.62, 14.52]**	**0.002**	0.92 [0.34, 1.48]
MAS						
T1	−**0.53 [**−**0.87,** −**0.18]**	−**0.94 [**−**1.24,** −**0.63]**	−0.41	[−0.87, 0.04]	0.074	0.22 [−0.31, 0.75]
T2	−**0.66 [**−**0.99,** −**0.34]**	−**1.0 [**−**1.29,** −**0.70]**	−0.33	[−0.77, 0.10]	0.132	0.11 [−0.37, 0.60]
T3	−**0.69 [**−**1.03,** −**0.35]**	−**1.26 [**−**1.56,** −**0.95]**	−**0.56**	**[**−**1.01,** −**0.11]**	**0.014**	0.37 [0.14, 0.89]
T4	−0.28 [−0.62, 0.05]	−**1.0 [**−**1.31,** −**0.69]**	−**0.72**	**[**−**1.18,** −**0.26]**	**0.002**	0.76 [0.20, 1.31]

*GMFM-66* gross motor function measure-66, *MAS* Modified Ashworth scale, *T1* 1-month data collection, *T2* 3-month data collection, *T3* 6-month data collection, *T4* 1-year data collection

The analysis of PEDI data showed that self-care (mean change 3.43, 95%CI 1.79 to 5.08) and mobility (mean change 3.88, 95%CI 1.48 to 6.27) dimensions as well as total PEDI score (mean change 8.53, 95%CI 4.98 to 12.08) increased significantly in the UCT-MSC group compared to baseline (Fig. 4C), and they were also significantly higher than the control group with large effect size (Cohen’s *d*> 0.5) (Table 3). Regarding CP-QoL, the mean changes in domains including friends and family, participation in activities, communication, and total scores were statistically higher than the control group with large effect size (Cohen’s *d*> 0.5) (Table 3), but there were no differences within groups that can show the changes were not clinically significant (Fig. 4D).

**Table 3:** The PEDI and CP-QoL mean difference within-groups (from baseline) and the difference between groups

Outcomes	Test of within-group mean change from effects of baseline		Test of between-groups effects mean change control group			
	Control	MSC	MSC vs control			
	Mean [95% CI]	Mean [95% CI]	***β***	[95% CI]	P.v	Cohen’s ***d*** [95% CI]
PEDI self-care						
T3	**1.89 [0.16, 3.62]**	**1.94 [0.39, 3.50]**	−0.01	[−2.36, 2.35]	0.99	0.01 [−0.5, 0.56]
T4	0.15 [−1.63, 1.95]	**3.43 [1.79, 5.08]**	**3.22**	**[0.75, 5.69]**	**0.011**	0.79 [0.22, 1.32]
PEDI mobility						
T3	0.13 [−1.69, 1.91]	1.63 [−0.62, 3.90]	1.53	[−1.38, 4.38]	0.29	0.33 [−017, 0.84]
T4	0.81 [−1.09, 2.72]	**3.88 [1.48, 6.27]**	**3.19**	**[0.16, 6.22]**	**0.039**	0.62 [0.07, 1.17]
PEDI social function						
T3	**3.04 [0.86, 5.23]**	**2.56 [1.01, 4.12]**	−0.52	[−3.30, 2.25]	0.71	0.09 [−0.60, 0.42]
T4	1.35 [−0.93, 3.60]	1.60 [−0.02, 3.23]	0.20	[−2.69, 3.09]	0.89	0.08 [−0.45, 0.62]
PEDI total						
T3	**5.09 [1.35, 8.83]**	**6.08 [2.68, 9.47]**	0.89	[−4.23, 6.02]	0.73	0.11 [−0.40, 0.62]
T4	1.58 [−2.30, 5.46]	**8.53 [4.98, 12.08]**	**6.87**	**[1.52, 12.21]**	**0.012**	0.70 [0.14, 1.23]
CPQOL						
Friends and family						
T3	−1.78 [−8.78, 5.20]	5.0 [−1.08, 11.09]	6.92	[−2.36, 16.22]	0.14	0.42 [−0.09, 0.93]
T4	−10.36 [−17.45, −3.28]	0.92 [−5.33, 7.18]	**11.23**	**[1.74, 20.71]**	**0.020**	0.63 [0.1, 1.16]
Participate in activities						
T3	−2.20 [−4.84, 0.43]	1.02 [−1.28, 3.33]	3.21	[−0.30, 6.73]	0.074	0.45 [−0.06, 0.97]
T4	−4.20 [−6.88, −1.53]	0.49 [−1.84, 2.87]	**4.61**	**[1.01, 8.20]**	**0.012**	0.65 [0.11, 1.19]
Communication						
T3	−2.65 [−4.42, −0.88]	−1.18 [−2.95, 0.59]	1.50	[−1.00, 4.00]	0.241	0.29 [−0.20, 0.78]
T4	−1.99 [−3.83, −0.15]	0.56 [−1.29, 2.43]	**2.54**	**[0-0.06, 5.16]**	**0.05**	0.63 [0.1, 1.16]
Physical health						
T3	−1.27 [−6.78, 4.29]	**6.85 [1.17, 12.52]**	**8.15**	**[0.25, 16.06]**	**0.043**	0.58 [0.06, 1.10]
T4	−3.54 [−9.12, 2.02]	2.0 [−3.82, 7.84]	5.34	[−2.71, 13.41]	0.19	0.40 [−0.12, 0.93]
Special equipment						
T3	−0.74 [−2.95, 1.42]	0.35 [−1.89, 2.59]	1.08	[−2.0, 4.21]	0.49	0.13 [−0.37, 0.64]
T4	0.45 [−1.76, 2.67]	−1.32 [−3.63, 0.97]	−1.71	[−4.91, 1.48]	0.29	0.31 [−0.84, 0.21]
Pain and impact of disability						
T3	−0.86 [−6.33, 4.59]	3.41 [−0.76, 7.59]	4.42	[−2.11, 11.36]	0.21	0.25 [−0.26, 0.76]
T4	1.31 [−4.22, 6.58]	3.52 [−0.77, 7.82]	0.70	[−4.55, 9.59]	0.48	0.16 [−0.36, 0.68]
Access to services						
T3	−6.09 [−11.43, −0.74]	−4.31 [−9.52, 0.89]	1.64	[−5.82, 9.11]	0.66	0.14 [−0.37, 0.65]
T4	−8.66 [−14.08, −3.24]	−4.93 [−10.28, 0.42]	4.0	[−03.61, 11.62]	0.30	0.24 [−0.28, 0.77]
Family health						
T3	−3.28 [−6.21, −0.35]	0.39 [−1.86, 2.64]	**3.66**	**[**−**0.5, 7.38]**	**0.054**	0.49 [−0.02, 1.01]
T4	−2.52 [−5.49, 0.44]	−1.09 [−3.40, 1.22]	1.29	[−2.49, 5.09]	0.503	0.21 [−0.31, 0.73]
CPQOL-total						
T3	−18.3 [−36.19, −0.37]	12.5 [−2.27, 27.23]	**30.74**	**[**−**0.24, 0.57]**	**0.010**	0.65 [0.13, 1.17]
T4	−29.3 [−47.5, −11.2]	0.05 [−15.11, 15.22]	**29.27**	**[5.50, 53.04]**	**0.016**	0.68 [0.14, 1.22]

### Secondary endpoint

The brain lesions in all participants were reported in Table 4. No significant improvements in the MRI of participants were observed compared to the baseline. The DTI analysis showed that mean FA increased significantly in the UCT-MSC group after 12 months of intervention (CST mean change: +0.032, 95%CI 0.02 to 0.03; PTR mean change: +0.024, 95%CI 0.020 to 0.028) and was statistically higher than the control group with large effect size (CST Cohen’s *d* 0.81 and PTR Cohen’s *d* 1.04) (Table 5). The mean MD decreased significantly in the experimental group after 12 months of intrathecal cell injections (CST mean change −0.035 × 10^-3^, 95%CI −0.04 × 10^-3^ to −0.02 × 10^-3^ ; PTR mean change −0.045 × 10^-3^, 95%CI −0.05 × 10^-3^ to −0.03 × 10^-3^) and was statistically lower than the control group with large effect size (CST Cohen’s *d* 0.77 and PTR Cohen’s *d* 0.55) (Table 5).

**Table 4:** The brain lesions of participants using magnetic resonance imaging

Findings	UCT-MSC group	Control group
Periventricular leukomalacia	32 (88.9%)	30 (83.3%)
Ventriculomegaly	17 (42.2%)	15 (41.7%)
Corpus callosum agenesis	6 (16.7%)	8 (22.2%)
Cerebral atrophy	2 (5.5%)	9 (25.0%)
Basal ganglia involvement	2 (5.5%)	4 (11.1%)
Focal ischemia	0 (0.0%)	2 (5.5%)
Cystic encephalomalacia	1 (2.8%)	2 (5.5%)
Porencephalic cyst	1 (2.8%)	1 (2.8%)

**Table 5:** Secondary endpoint analysis

Outcome	UCT-MSC (***n***=36)	control (***n***=36)
**Fractional anisotropy**		
**Corticospinal tract**		
12 months mean (SD)	0.48 (0.04)	0.42 (0.06)
Mean changes from baseline (95% CI)	0.032 (0.02 to 0.03)	−0.007 (−0.010 to −0.002)
Difference vs control (95% CI)	0.055 (0.02 to 0.08)	–
Cohen’s d (95%CI)	0.81 (0.36 to 1.26)	–
**Posterior thalamic radiate**		
12 months mean (SD)	0.37 (0.03)	0.32 (0.04)
Mean changes from baseline (95% CI)	0.024 (0.020 to 0.028)	−0.020 (−0.02 to −0.01)
Difference vs control (95% CI)	0.059 (0.3 to 0.8)	–
Cohen’s *d* (95%CI)	1.04 (0.68 to 1.41)	–
**Mean diffusivity (×10**^**3**^)^*^		
**Corticospinal tract**		
12 months mean (SD)	0.93 (0.05)	1.00 (0.12)
Mean changes from baseline (95% CI)	−0.035 (−0.04 to −0.02)	0.040 (0.02 to 0.05)
Difference vs control (95% CI)	−0.07 (−0.11 to −0.02)	–
Cohen’s *d* (95%CI)	0.77 (0.26 to 1.28)	–
**Posterior thalamic radiate**		
12 months mean (SD)	1.02 (0.07)	1.09 (0.19)
Mean changes from baseline (95% CI)	−0.045 (−0.05 to −0.03)	0.033 (0.01 to 0.05)
Difference vs control (95% CI)	−0.07 (−0.15 to −0.003)	–
Cohen’s *d* (95%CI)	0.55 (0.01 to 1.11)	–

*The mean (SD) should be divided by 1000

### Safety endpoint

There were 5 adverse events that were identified in participants. Two cases in the UCT-MSC group (2/36 or 5.5%) experienced fever (38 to 38.7^o^C) within 24 h of intrathecal cell injections but resolved spontaneously and with no diagnosis of infection (e.g., meningitis). Mild to moderate irritability (*n*= 3 control group and *n*= 6 UCT-MSC group), headache (*n*= 1 control group and *n*= 5 UCT-MSC group); low back pain (*n*= 8 UCT-MSC group); and vomiting (*n*= 1 UCT-MSC group) were other reported adverse events within 24 h after procedures. These primary events were recorded by nurses and medical doctors who were not informed about the study. These data were not shared with the investigators until the end of the study. All symptoms were resolved, and no serious adverse events were reported during the follow-up periods.

## Discussion

This study assessed the safety and efficacy of intrathecal administration of a single dose of the UCT-MSC in children with CP in referral hospitals. The data of GMFM-66, PEDI, CP-QoL, and MAS scores showed that cell therapy was clinically effective. The data of DTI also showed significant improvements in white matter structural integrity of cases treated with stem cells. The intrathecal injection of the cells was safe in participants, and there was no difference in serious adverse events compared to the control group.

Motor impairments are the most common disability symptoms in CP. Our study showed that intrathecal injection of the UCT-MSC improved motor function significantly after 6 months of cell injection compared to the control group. The improvements remained until the end of follow-up visits. One non-randomized trial on 8 pairs (16 individuals) of identical twins reported that gross motor function had no significant improvements after 1 month of 4–6 × 10^7^ allogenic UCT-MSC intrathecal injection but significant improvements were observed after 6 months. Hereditary factor was reported to be a predictor of efficacy [40]. Our study showed that the mean GMFM-66 scores decreased and MAS increased in the experimental group at the last follow-up visit (1 year) compared to the previous one (6 months). This may show the temporary effects of cell transplantations in CP. Increasing injection frequency may improve the efficacy in longer periods. The overall changes in the mean GMFM-66 scores in both groups of our trial were lower compared to the literature [41–43]. This can be due to the baseline characteristics including the age of participants. The mean age of our included cases was higher than prior studies [41–43]. It has been shown that the potential of growth motor in children with CP plateaus after 5 to 7 years of age [44], and we could expect better treatment responses in individuals with ages closer to the timing of the injury [21]. Furthermore, the lower mean change in the treatment group could also be due to the single-dose injection of UCT-MSCs.

Emerging evidence showed that stem cells derived from different sources could be plausible treatments to improve motor function in people with CP. A recent randomized clinical trial reported that four intrathecal injections of autologous bone marrow MSC improved gross and fine motor functions compared to the placebo [41]. It should be noted that cord cell transplant is generally better-suited in children and adolescents as cells can be collected safely and without invasive and painful procedures. The use of allogenic umbilical cord cells is also cheaper and less time-consuming than the use of autologous bone marrow cells. The separation, purification, expansion, characterization, and harvest of MSCs from the bone marrow could take much longer time (about 1 month) than UCT-MSCs [40]. Other clinical studies used bone marrow mononuclear cells [45, 46] or MSC [47] reported significant functional improvements. The human embryonic tissue is another source of stem cells that was shown to be safe and effective in individuals with CP [48]. This source, however, cannot be used in many clinical conditions due to difficulties in isolation methods and requirement for a complex culture system [49]. Furthermore, harvesting of cells can lead to death of embryo that raises major ethical concerns. Neural progenitor cells and olfactory ensheathing cells were other effective cell types used in children with CP and isolated from aborted human fetuses [50, 51].

Stem cells can be administered by different routes. The most appropriate route was, however, unknown. Several studies reported that intravenous or intra-arterial infusions of stem cells can accelerate the functional development of people with CP [52–54]. Although cell transplant by these routes is less invasive than intrathecal cell injection; studies showed that fewer cells than expected could reach to the lesion areas using intra-arterial/intravenous deliveries due to the possible retaining by other organs or inability of cells to cross the blood brain barrier [55, 56]. Intra-arterial cell infusions were also showed to be associated with increased risk of micro-embolic events [57]. One case-report used the combination of intravenous and intrathecal deliveries of UCT-MSC and reported obvious improvements in EEG, motor function, and language expression [58]. Intra-ventricular route of cell administration was also suggested but is more invasive and cannot be used generally in all patients [50].

The data of quantitative DTI can be so helpful to measure the alterations of white matter integrity after cell transplant. To date, few studies assessed the changes in DTI parameters after cell therapy in different disorders [59–61]. The ROI-based analysis of CST in individuals with CP reported significant reductions in FA [62–66] and increased MD [67] compared to the healthy individuals. It was shown that GMFCS was associated with the FA of CST when assessing ROI-based fractional anisotropy [62, 64, 65, 68]. Some studies reported significant FA reductions in PTR of cases with CP [62, 69]. The ROI-based fractional anisotropy of PTR was also shown to be correlated with the GMFCS levels [62, 68, 69]. Our results showed that cell transplant increased FA and decreased MD of both CST and PTR significantly. These changes can show that the diffusion of water molecules is constrained in the CST and PTR after cell therapy that could be associated with increased rate of myelination and improvements in white-matter structural integrity.

This trial had different strengths. To our knowledge, this is the first clinical trial used quantitative DTI after intrathecal cell injection in individuals with CP. Randomization and blinding were other main strengths of this study. The multi-center prospective population-based approach of this trial enhanced the external validity of the results. There were also some limitations to our study. Single and fixed dose of UCT-MSC was administered in our participants. Repeated injections based on weights of individuals might increase the efficacy of the cells. Further studies should compare different routes and sources of stem cells to find the most appropriate protocol of cell transplant. The optimal dose of cells can be identified by conducting cross-over studies. Longer follow-up periods are suggested to realize the long-term efficacy of cell transplant. Using other imaging data including magnetic resonance spectroscopy can also be useful to better understand the underlying mechanisms of stem cells in neuronal repair.

## Conclusions

The intrathecal injection of the UCT-MSC may be safe in children diagnosed with CP and improve the clinical and imaging outcomes.

## Supplementary information

**Additional file 1: Supplement 1.** Levels of gross motor function classification system (GMFCS).

**Additional file 2: Supplement 2.** Modified Ashworth scale

**Additional file 3: Supplement 3.** Sample size calculation

**Additional file 4: Supplement 4.** CONSORT 2010 checklist of information to include when reporting a randomised trial*

## Data Availability

The datasets generated and/or analyzed during the current study are not publicly available but are available from the corresponding author on reasonable request.

## Abbreviations

ANOVA: Analysis of variance
CI: Confidence interval
CNS: Central nervous system
CP: Cerebral palsy
CP-QoL: Cerebral palsy quality of life
CST: Corticospinal tract
DMEM: Dulbecco’s modified Eagle’s medium
DTI: Diffusion tensor imaging
EEG: Electro-encephalography
FA: Fractional anisotropy
GEE: Generalized estimating equations
GMFCS: Gross motor function classification system
GMFM: Gross motor function measure
LAL: Limulus amebocyte lysate
MD: Mean diffusivity
MAS: Modified Ashworth scale
MRI: Magnetic resonance imaging
PEDI: Pediatric evaluation of disability inventory
PTR: Posterior thalamic radiation
ROI: Region of interest
SD: Standard deviation
SEM: Standard error of the mean
UCT-MSC: Umbilical cord tissue mesenchymal stem cell
