# Supplementary material for: Clinical and imaging outcomes after intrathecal injection of umbilical cord tissue mesenchymal stem cells in cerebral palsy: a randomized double-blind sham-controlled clinical trial
Source: Stem Cell Res Ther. 2021 Aug 6;12:439. doi: 10.1186/s13287-021-02513-4 (PMC8343813; doi:10.1186/s13287-021-02513-4)
Supplement: Supplementary file 1 — Additional file 1: Supplement 1. Levels of gross motor function classification system (GMFCS). [file 13287_2021_2513_MOESM1_ESM.docx]

**Supplement 1.** Levels of gross motor function classification system (GMFCS).

- **Level I:** Decreased speed, balance, and coordination but normal walking indoors and outdoors and climbing stairs without using any support and performing usual activities (e.g. running and jumping)
- **Level II:** Walking difficulties on long distances and balancing on uneven surfaces, inclines, or in crowds. May climb stairs by using railing.
- **Level III:** Walking in most indoor settings by using hand-held mobility device. Walking on long distances and uneven surfaces by using wheeled mobility. May climb stairs by using railing with supervision or assistance.
- **Level IV:** Limited walking ability and using wheelchair in most settings. May stand with or without assistance.
- **Level V:** No walking ability and using wheelchair in all settings. Cannot stand even with assistance. No ability of maintaining head and trunk postures against gravity.
