# Supplementary material for: Clinical and imaging outcomes after intrathecal injection of umbilical cord tissue mesenchymal stem cells in cerebral palsy: a randomized double-blind sham-controlled clinical trial
Source: Stem Cell Res Ther. 2021 Aug 6;12:439. doi: 10.1186/s13287-021-02513-4 (PMC8343813; doi:10.1186/s13287-021-02513-4)
Supplement: Supplementary file 2 — Additional file 2: Supplement 2. Modified Ashworth scale [file 13287_2021_2513_MOESM2_ESM.docx]

**Supplement 2.** Modified Ashworth scale

**0:** No increase in muscle tone (normal)

**1:** Slight increase in muscle tone, manifested by a catch and release or by minimal resistance at the end of the range of motion when the affected part(s) is moved in flexion or extension

**1+ (2):** Increase in muscle tone, manifested by a catch, followed by minimal resistance throughout the remainder (less than half) of the range of motion

**3:** More marked increase in muscle tone through most of the range of motion, but affected part(s) easily moved

**4:** Considerable increase in muscle tone, passive movement difficult

**5:** Affected part(s) rigid in flexion or extension
