# Supplementary material for: Clinical and imaging outcomes after intrathecal injection of umbilical cord tissue mesenchymal stem cells in cerebral palsy: a randomized double-blind sham-controlled clinical trial
Source: Stem Cell Res Ther. 2021 Aug 6;12:439. doi: 10.1186/s13287-021-02513-4 (PMC8343813; doi:10.1186/s13287-021-02513-4)
Supplement: Supplementary file 3 — Additional file 3: Supplement 3. Sample size calculation [file 13287_2021_2513_MOESM3_ESM.docx]

**Supplement 3.** Sample size calculation

F tests - ANOVA: Repeated measures, between factors

Analysis: A priori: Compute required sample size

Input: Effect size f = 0.25

α err prob = 0.05

Power (1-β err prob) = 0.80

Number of groups = 2

Number of measurements = 4

Corr among rep measures = 0.5

Output: Noncentrality parameter λ = 10.2000000

Critical F = 3.0882396

Numerator df = 2.0000000

Denominator df = 99.0000000

Total sample size = 72

Actual power = 0.8110661

…………...................................................................................................................

F tests - ANOVA: Repeated measures, between factors

Analysis: Post hoc: Compute achieved power

Input: Effect size f = 0.3540745

α err prob = 0.05

Total sample size = 72

Number of groups = 2

Number of measurements = 4

Corr among rep measures = 0.5

Output: Noncentrality parameter λ = 21.6637203

Critical F = 3.0828520

Numerator df = 2.0000000

Denominator df = 105

Power (1-β err prob) = 0.9889684
